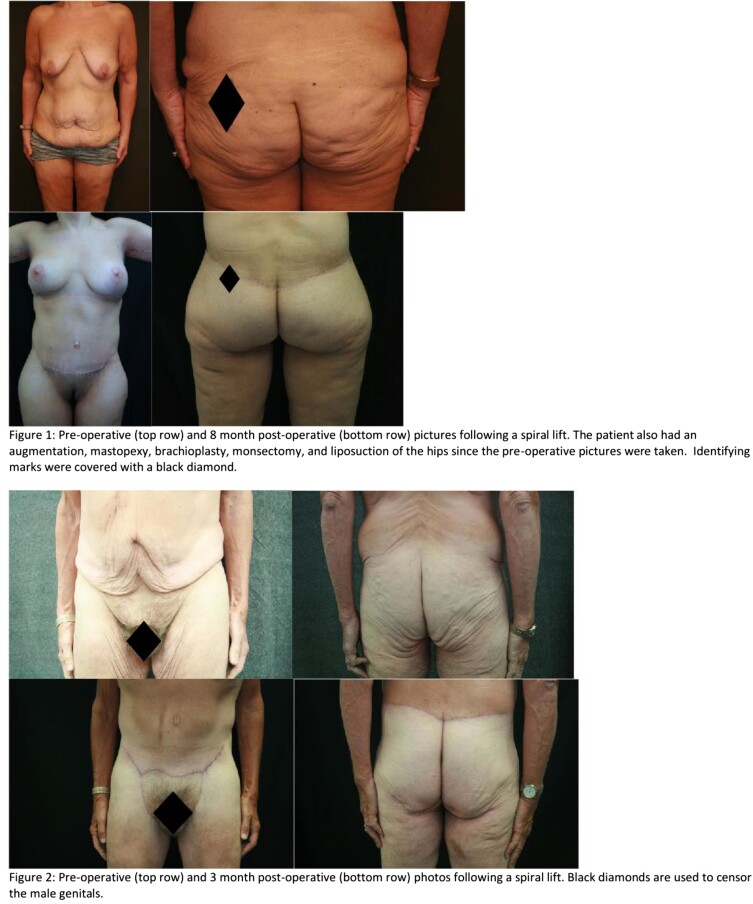# The Spiral Lift: A Novel Twist on Body Contouring

**DOI:** 10.1093/asjof/ojad027.019

**Published:** 2023-04-14

**Authors:** Lauren Woldanski, Robert Russell

**Affiliations:** Southern Illinois University, Springfield, IL; Heartland Plastic Surgery, Springfield, WI

## Abstract

**Goals/Purpose:**

Thigh lifts historically have been performed using a vertical excision to correct the horizontal laxity.^1^However, this leaves an unsightly scar when wearing shorts or a bikini bottom. More recently the horizontal scar saving thighplasty has been described, but this only addresses a limited medial portion of the thigh laxity.^2^While plastic surgeons perform abdominoplasty concurrently with medial thigh lifts,^3^ there is no described technique of this type of spiral lift in the literature. The spiral lift does not use liposuction of the medial thighplasty portion of the procedure as is used in many of the published techniques.^4^ Moreover, compared to other described horizontal thigh lift techniques, which use a much smaller horizontal incision,^5^ the spiral lift extends this incision in the groin crease and into the inferior gluteal crease, resulting in a longer but very well hidden incision. This provides maximal lift of the entire thigh while avoiding a vertical scar. Our goal was to develop a technique to lift the medial and lateral aspect of the leg and the buttock skin while also minimizing visible scarring.

**Methods/Technique:**

The lower abdominoplasty/belt lipectomy incision is designed in the pre-operative area such that the post-operative incision will be hidden in a pair of underwear of choice. The thighplasty incisions are designed in the groin crease, continuing into the lower abdominoplasty incision, and extend posteromedially around into the inferior gluteal crease. A pinch test is used to determine the appropriate amount of tissue that can be excised. The patient is intubated on the stretcher, then flipped into the prone position on an OR bed. Excision is performed to the level of the thoracolumbar fascia on the back and Scarpa’s fascia for the thighs to avoid the posterior cutaneous nerve. Two drains are left in the back. Following closure, the patient is flipped supine onto a second OR bed. A standard abdominoplasty is then performed in continuation with the back incision to complete the belt lipectomy. The anterior medial thigh excision is again performed at the level of Scarpa’s fascia, staying superficial in the region of the femoral vessels and lymphatics. Two drains are placed anterior in the abdomen.

**Results/Complications:**

A total of 12 pts have undergone a full belt lipectomy with the spiral lift thighplasty since it first was performed in July 2019. This technique has shown good cosmetic results (Figures 1&2), providing a circumferential vertical lift of the buttock and entire thigh while also addressing the abdomen and back. We have had good success in both men and women. This procedure is especially beneficial for massive weight loss patients, but we have also done the spiral thigh lift with abdominoplasties not requiring circumferential body lift. Five patients also underwent additional concurrent procedures involving the breast (mastopexy, augmentation, etc). Some patients had same day surgery (7), while five were observed overnight. The patients do tend to get some mild swelling of the mons. Scrotal swelling can be minimized with scrotal elevation and compression. Three patients had dehiscence of the thigh wounds, two of which were minor and were managed with dressing changes. One was caused by a fall at home and required return to the operating room for repair, but also had subsequent dehiscence requiring dressing changes. This was one of two patients that were re-admitted following the procedure, the other being a patient admitted for UTI sepsis 10 days after surgery. Forty-two percent of patients have had minor revisions following the procedure, 80% of which are performed concurrently with another primary surgery (eg. brachioplasty, cervicoplasty). One patient experienced transient lymphedema of the bilateral thighs. We have experienced no major infections. Some patients have experienced descent of the anterior medial thigh incision, visible in bikini bottoms.

**Conclusion:**

The spiral lift is a technique that can successfully achieve a full body lift and address the thigh laxity circumferentially while completely hiding the scars in the underwear lines or natural skin creases.

**Figure:**